# Whole-Genome Sequence of Multidrug-Resistant *Pseudomonas aeruginosa* Strain BAMCPA07-48, Isolated from a Combat Injury Wound

**DOI:** 10.1128/genomeA.00547-16

**Published:** 2016-07-07

**Authors:** Fatemeh Sanjar, S. L. Rajasekhar Karna, Tsute Chen, Ping Chen, Johnathan J. Abercrombie, Kai P. Leung

**Affiliations:** a Dental and Craniofacial Trauma Research and Tissue Regeneration Directorate, U.S. Army Institute of Surgical Research, JBSA Fort Sam Houston, Texas, USA; b The Forsyth Institute, Cambridge, Massachusetts, USA

## Abstract

We report here the complete genome sequence of *Pseudomonas aeruginosa* strain BAMCPA07-48, isolated from a combat injury wound. The closed genome sequence of this isolate is a valuable resource for pathogenome characterization of *P. aeruginosa* associated with wounds, which will aid in the development of a higher-resolution phylogenomic framework for molecular-guided pathogen-surveillance.

## GENOME ANNOUNCEMENT

The rise of multidrug resistant (MDR) organisms in war wounds is of major concern to the health care community. Efforts are increasing to identify and characterize genotypes of war wound MDR pathogens for improved biosurveillance, wound-care response, and outcome (1–4). The Gram-negative opportunistic pathogen, *Pseudomonas aeruginosa*, is ubiquitously found in the environment (5, 6), exhibits intrinsic antibiotic resistance profile (7, 8), and is considered one of the primary etiological agents of wound and nosocomial infections (9–11). *P. aeruginosa* strain BAMCPA07-48 was kindly provided by San Antonio Military Medical Center (SAMMC) and was isolated from a wound caused by combat injury. Antimicrobial susceptibility testing (AST) of BAMCPA07-48 revealed resistance to antibiotic agents including fluoroquinolone, tetracycline, vancomycin, zeocin, trimethoprim, nitrofurantoin, aminoglycoside, and a wide range of β-lactams; susceptibility was only observed with tobramycin treatment (≥4 µg/mL).

The BAMCPA07-48 genomic DNA was isolated from overnight culture using a Wizard Genomic DNA purification kit (Promega). Genomic DNA of BAMCPA07-48 was subjected to whole-genome sequencing using a hybrid sequencing method with PacBio and Illumina HiSeq platforms (12, 13). With 200× coverage, the draft genome of BAMCPA07-48 was assembled and closed using BGI hybrid assembler (14). Genome annotation and visualization was conducted using GeneMarkS+ (15), resulting in the identification of 6,739 genes and 64 tRNA and 12 rRNA molecules. The genome sequence of BAMCPA07-48 revealed a G+C content of 66.03% and a genome size of 7.0 Mb.

Availability of high-quality closed-genome sequence enables rapid determination of virulence-state and phylogenomic profiling according to well-established genotypic classification methods using *in silico* computational approaches (16–20). The use of high-throughput next generation sequencing for whole-genome sequence analysis of BAMCPA07-48 will aid in high-resolution genotypic characterization and virulence-profiling while investigating the pathogenome evolution of this strain in relation to sequenced *P. aeruginosa* extant genotypes (21–24). Extending on and confirming our initial *in vitro* AST study, sequence-based analysis of the BAMCPA07-48 strain using an antibiotic resistance gene identifier (RGI) (25) revealed carriage of resistance genes against a wide range of antimicrobial classes including aminoglycoside, fosfomycin, triclosan, rifampin, trimethoprim, tetracycline, aminocoumarin, macrolide, β-lactams, chloramphenicol, polymyxin, and fluoroquinolone. Multilocus sequence typing (MLST) (16, 17) placed BAMCPA07-48 as sequence type 313 (26) and motility typing (*fliC*) classified it as a type A and *flaG*-positive strain (27–29). Further studies remain necessary to better understand genotypic variations and different virulence profiles present in *P. aeruginosa* isolates associated with combat injury wounds and when compared to extant genotypes.

In contrast to draft genomes, a complete genome sequence provides a higher-resolution finished product in which the order and accuracy of each single base pair of the genome is verified and there are no gaps in the genome (24, 30). The complete genome sequence of BAMCPA07-48 enables accurate downstream functional genomics analysis, precise identification of its genetic organization, and future comparative genomic studies with relation to the growing collection of sequenced *P. aeruginosa* strains.

### Nucleotide sequence accession number.

The complete genome sequence for *P. aeruginosa* strain BAMCPA07-48 has been deposited in GenBank under the accession number CP015377.
